# Roadblock: improved annotations do not necessarily translate into new functional insights

**DOI:** 10.1186/s13059-021-02542-5

**Published:** 2021-11-22

**Authors:** Nicola A. L. Hall, Becky C. Carlyle, Wilfried Haerty, Elizabeth M. Tunbridge

**Affiliations:** 1 Department of Psychiatry, University of Oxford, Oxford, UK; 2 Oxford Health NHS Foundation Trust, Oxford, UK; 3 Massachusetts General Hospital, Boston, USA; 4 The Earlham Institute, Norwich, UK; 5 School of Biological Sciences, University of East Anglia, Norwich, UK

The advent of cost-effective high-throughput nucleotide sequencing means that information about the transcriptome is accruing at an exponential rate, rapidly refining our understanding of the diversity of gene products. It is important that these findings are readily accessible to the wider scientific community to maximise their impact. However, there are multiple barriers to their efficient dissemination and their translation into functional insights. Here, we outline how the status quo can result in information becoming siloed and/or ambiguous, using the *CACNA1C* gene, which encodes a voltage-gated calcium channel, as an example. We highlight three areas that pose potential barriers to effective information transfer and offer suggestions as to how these may be addressed: firstly, a lack of clarity about the strength of the evidence for individual transcripts in current annotations; secondly, limitations to the transfer of information between nucleotide and protein databases; thirdly, challenges relating to the nomenclature used for transcriptional events and RNA modifications, both for genomic researchers and the wider scientific community.

## How reliable are current transcriptomic annotations?

Many projects have produced, or are aiming to produce, a reference transcriptome to synthesise the wealth of (highly redundant) sequencing information, although the resulting annotations vary due to differences in algorithms applied and the extent to which annotations are manually curated [1]. However, although annotations continue to improve, inaccuracies are introduced by the need to computationally reconstruct full-length transcript isoforms from short-read data [2]. Thus, it is possible that some currently annotated full-length isoforms are either incomplete or represent false positives [3]. Conversely, biases in the types of samples that have been historically sequenced mean that false negatives, i.e. transcripts that exist but are not currently annotated, are also likely. Technical biases can be introduced by sample preparation [4]. However, even if it were possible to prepare perfect sequencing libraries, annotations are inherently biased by the relative unavailability of many types of relevant input material, particularly in the case of human tissues. For example, in the case of human brain, outside of rarefied cellular populations [5], large-scale sequencing efforts necessarily focus either on bulk tissue, which contains a mixture of diverse cellular populations, or single nucleus sequencing, which does not necessarily reflect the total transcript pool [6].

Novel long-read RNA sequencing technologies, such as Oxford Nanopore Technologies and PacBio, allow full length transcript isoforms to be sequenced, thereby providing the potential to eliminate false positive isoforms arising from reconstruction errors. In addition, sequencing at depth and/or combining this technology with enrichment approaches also provides a means to identify novel, full-length transcripts. For example, targeted long-read sequencing of *CACNA1C* transcripts from just one start exon identified 38 novel exons and 241 novel transcript isoforms, as well as abundant splice site variations [7]. As the use of long-read sequencing becomes more prominent it is likely that many novel exons and isoforms will be discovered for other genes [8]. Clearly, it is possible that many of the minor isoforms reflect transcriptional noise. However, it is also possible that transcripts that appear minor in studies of bulk tissue are more prominent in cellular subpopulations. In support of this assertion, ~ 90% of the population of *CACNA1C* transcripts sequenced in human brain are predicted to encode functional voltage-gated calcium channels (i.e. they predict full length channels that include all domains critical for function) [7]. This is far higher than would be expected if they simply represented transcriptional noise, which would, by definition, be expected to induce frame shifts in two thirds of transcripts. Long-read sequencing studies may also require current annotations to be re-evaluated to remove false positives. Despite detecting a total of 251 different *CACNA1C* isoforms, there was strong support for only 10 of the 31 previously annotated in GENCODE (v27) and only one of these was amongst the ten most abundant isoforms. It is likely that some of the annotated isoforms that were not found in adult brain are expressed in other tissues and/or at other stages of development and ageing, but some may be false positives.

Thus, current annotations, even those generated using RNA-seq data, remain far from complete. The impact of inaccuracies in reference annotations is far-reaching since they are frequently used for mapping RNA-Seq and, in some instances, proteomic data. Against this backdrop, long-read sequencing has significant potential to improve annotations, particularly in combination with targeted approaches. As annotations begin to incorporate long-read sequencing data it would be extremely valuable if individual transcripts and splicing events could be flagged as being either predicted, based on reconstruction from short-read data, or validated, by long read sequencing, mass-spectrometry peptide identification, or other approaches, to help researchers to determine the strength of the underlying evidence for specific isoforms and to select a reference that suits their needs. For example, in ‘omics’ level proteomics, peptide identification is generally performed by matching peptide fragment products to a reference, meaning this process is a fine balance between the complexity of the reference and the number of multiple tests performed. There are therefore significant advantages to having a choice between a streamlined, high confidence transcript reference, and a more experimental comprehensive reference, depending on experimental goals.

## Bridging the gap between the gene annotations and function

A primary reason for generating high-quality transcriptomic annotations is to inform functional studies of gene products. For example, in the case of *CACNA1C*, splicing events across the gene have been shown to influence multiple aspects of channel function [9], resulting in the production of channels tuned to the needs of the tissue type in which they are expressed [10]. However, the historical lack of information about the structure of full-length channel isoforms made it largely impossible to study native isoforms, nor to understand how different splicing events might interact with one another. This information is not only important to understanding the function of these channels *in vivo* but is also of medical relevance, given that splicing modulates the clinical presentation of Timothy Syndrome, a severe developmental condition caused by *CACNA1C* mutations [11] and because there is interest in developing novel calcium channel blockers for psychiatric indications that can selectively target brain channel isoforms [12].

It is tacitly assumed that improved transcriptomic annotations will automatically feed into functional studies [13]; however, our experience is that in practice this does not necessarily occur, due to the different sources of information used by different disciplines. Researchers studying protein structure and function rely largely on information in the Uniprot and the Protein Databank (PDB) protein databases and the scientific literature, since nucleotide-centred browsers are poorly suited for visualising and annotating proteins. Notably, there are significant gaps in information transfer between transcriptomic and protein annotations. For example, 10 of the 32 full-length *CACNA1C* transcripts annotated in Ensembl lack corresponding protein entries in Uniprot (see Table 1). This barrier to information flow occurs in both directions: Uniprot contains four manually curated full-length *CACNA1C* protein isoforms with a 29 amino acid N-terminal truncation (Q13936-16, -17, -18 and -28) that is not encoded by any of the current full-length Ensembl isoforms (Table 1). These discrepancies likely result from the sources of information used to generate these distinct databases. Uniprot incorporates information from direct protein sequencing, the PDB and the scientific literature, as well as translated coding sequences derived from primary sequencing data obtained from the International Nucleotide Sequence Database Collaboration (INSDC). Although Uniprot entries may include information from computationally assembled annotations, such as Ensembl, these sequences are not automatically included. Conversely, although information from Uniprot is used to refine Ensembl annotations [14], sequence information from Uniprot does not directly get incorporated into these annotations. Thus, although efforts are made to try and link the protein and nucleotide sequence information repositories, there remain significant differences between them. The need for different interfaces for interacting with nucleotide and protein databases will likely remain, given the differing needs of the communities that they serve, but substantially improved synchronisation and cross-referencing between them is required to maximise their utility.

**Table 1:** Discrepancies in the annotations of full-length *CACNA1C*/Ca_V_1.2 isoforms between Ensembl and Uniprot

Ensembl ID^a^	Nucleotide length (bp)	Uniprot ID^a^	Amino acid length
ENST00000399655.6	13744	Q13936-12	2138aa
ENST00000347598.9	13888	Q13936-11	2186aa
ENST00000327702.12	13849	A0A0A0MR67	2173aa
ENST00000399603.6	13744	Q13936-37	2138aa
ENST00000399641.6	13744	Q13936-23	2138aa
ENST00000399617.6	7719	A0A0A0MSA1	2173aa
ENST00000344100.7	6634	Q13936-14	2179aa
ENST00000399638.5	6595	Q13936-31	2166aa
ENST00000399606.5	6571	Q13936-30	2158aa
ENST00000399621.5	6568	Q13936-24	2157aa
ENST00000399637.5	6568	Q13936-27	2157aa
ENST00000402845.7	6568	Q13936-13	2157aa
ENST00000399629.5	6562	Q13936-32	2155aa
ENST00000399591.5	6535	Q13936-29	2146aa
ENST00000399595.5	6535	Q13936-25	2146aa
ENST00000399649.5	6529	Q13936-15	2144aa
ENST00000399597.5	6511	Q13936-22	2138aa
ENST00000399601.5	6511	Q13936-20	2138aa
ENST00000399644.5	6511	Q13936-21	2138aa
ENST00000682835.1	8541	-	2138aa
ENST00000406454.8	8166	F5GY28	2209aa
ENST00000399634.6	8133	E9PDI6	2198aa
ENST00000335762.10	7411	F5H522	2163aa
ENST00000682462.1	7059	-	2168aa
ENST00000682544.1	7047	-	2251aa
ENST00000683482.1	6889	-	2135aa
ENST00000683781.1	6750	-	2168aa
ENST00000683824.1	6582	-	2193aa
ENST00000683956.1	6553	-	2168aa
ENST00000683840.1	6553	-	2168aa
ENST00000682686.1	6478	-	2127aa
ENST00000682336.1	6459	-	2152aa
-	-	Q13936	2221aa
-	-	Q13936-2	2257aa
-	-	Q13936-3	2221aa
-	-	Q13936-4	2201aa
-	-	Q13936-5	2201aa
-	-	Q13936-6	2193aa
-	-	Q13936-7	2193aa
-	-	Q13936-8	2210aa
-	-	Q13936-9	2222aa
-	-	Q13936-10	2240aa
-	-	Q13936-16	2144aa
-	-	Q13936-17	2109aa
-	-	Q13936-18	2180aa
-	-	Q13936-19	2127aa
-	-	Q13936-26	2139aa
-	-	Q13936-28	2169aa
-	-	Q13936-33	2173aa
-	-	Q13936-34	2251aa
-	-	Q13936-35	2135aa
-	-	Q13936-36	2173aa

^a^Isoform information obtained from https://www.ensembl.org/ and https://www.uniprot.org/ on 14th October 2021. Note that only isoforms predicted to encode functional calcium channels are included

## What’s in a name? Harmonising nomenclature across databases and the literature

New exons and isoforms will continue to be discovered as sequencing breadth and depth increase. Furthermore, future annotations will also need to capture details of the RNA (and protein) modifications that are being identified by novel technological approaches, such as direct RNA sequencing [15]. Incorporating information about novel exons into transcriptomic annotations is relatively straightforward: exons are typically numbered from 5’ to 3’ along a gene and renumbered as needed, since they are directly linked to their chromosomal location. However, exon renumbering causes significant problems for researchers studying the functional impact of splicing. For example, the functional impact of *CACNA1C* splicing is well studied and much information predates transcriptomic annotations [9, 10]. Thus, whilst generic exon-specific nomenclature exists (e.g. Ensembl’s ENSE references), it is not widely used by the calcium channel community. Instead, a field-specific naming schema has evolved that uses the protein model, rather than transcriptomic annotations, as its basis (9). Changes to this, albeit haphazard, naming schema have the potential to cause substantial confusion. Indeed, a specific example has already occurred. There are inconsistencies in the naming of two alternatively spliced exons in *CACNA1C*, which are functionally important and the locations of Timothy syndrome mutations. In some publications, they are named Exons 8 and 8A [16, 17], whilst other publications use 8A and 8B [9]; as a result, “8A” can refer to either of the two mutually exclusive exons, depending on context, and is therefore a common source of confusion in the field. A further complexity to the naming (and renaming) of exons comes from the presence of novel splice junctions in exons. For example, *CACNA1C* contains multiple splice sites within exons that lead to small-scale (2–5 amino acid) changes in peptide sequence [7]. To our knowledge, none of the existing nomenclature captures such nuanced events; instead, exons containing alternative splice sites are typically broken up into discrete but contiguous exonic parts [18]. Despite their small scale, variation of this type can significantly alter protein function, as has been demonstrated in the case of *CACNA1C* [16], and so will need to be captured within any novel naming schema.

Using genomic co-ordinates to disambiguate RNA and protein isoforms, RNA modifications, exons and genomic loci is one possible solution. However, current genomic co-ordinates will likely have to change as long-read DNA sequencing increasingly uncovers the ‘dark’ areas of the genome, such as tandem repeat elements [19]. Alongside the drive to sequence a larger number and greater diversity of complete genomes, these advances challenge the current concept of a single reference genome per organism [20] Thus, the complexities associated with moving from the concept of a single reference genome to something more representative of species diversity will have knock-on effects for annotations, particularly where isoforms, modifications, exons, or other features are genotype dependent. Furthermore, some RNA modifications are isoform-specific [15] and must therefore be mapped to transcriptomic annotations, rather than directly to whichever genomic standard is adopted. Future annotations will therefore need to ensure that information is mapped at the relevant level, be that genome, transcriptome or proteome.

## Concluding remarks

Our experiences highlight the challenges in ensuring that improvements in transcriptomic annotations are translated into novel biological insights. Central to this problem is the relative lack of information flow between existing databases. This problem will only be exacerbated by emerging improvements in our understanding of the nuances of the transcriptome. As others have highlighted, in coming years as more individual genomes are sequenced it will be necessary to reappraise our understanding of what we mean by the ‘reference genome’ [20]. We would advocate going further: to maximise the impact of emerging technologies, we will need to put robust systems in place to ensure that information is accurately recorded at the appropriate level—be this genomic, transcriptomic or proteomic—and that it is able to flow effectively between these related but distinct annotations. For example, the identification of a high-confidence peptide sequence spanning a splice junction provides an orthogonal source of support for such events in transcriptomic annotations. Critically, to maximise their impact, the annotations of the future will need to be effectively collated and referenced in a manner sensitive to the needs of the different groups of end users, as well as being harmonised with the existing scientific literature. We provide some suggestions for steps that can be taken to work towards the goal of future-proof annotations (Table 2); however, such efforts will be successful only if widely agreed upon and used across the whole scientific community. We therefore advocate that conversations about how best to capture and collate this information in an accessible and searchable format engage with as wide a group of scientists as possible. The appropriate curation of data will be crucial to the successful and efficient translation of information; however, the infrastructure for effective data management and curation is an area that has been severely neglected [23]. We there conclude by calling for science funders to prioritise this vital activity, since the status quo limits the impact of the wealth of data being generated.

**Table 2:** Towards future-proof annotations

To ensure that improved annotations lead to meaningful insights into biological function, it is essential that they are accurate, user friendly and sympathetic to the needs of the end user. As we highlight in the main text, the status quo means that information does not readily flow between protein and transcriptomic annotations, limiting its uptake by those undertaking functional studies. The reach of future annotations is likely to maximised by engaging with the widest possible community of scientists in the process of the development of updated annotations, both to ensure that they are fit for purpose and to identify potential solutions from other fields (see point 1 for an example). Here, we highlight some initial suggestions going forwards based on our experience, with the aim of starting a dialogue with the wider scientific community. *Genomic annotations:* 1. The standard reference genome will likely need to adapt to incorporate human genomic diversity as more individuals are sequenced. It may be helpful to consider moving away from the use of ‘genomic coordinates’ to ‘genomic space’: a set of ‘averaged’ coordinates onto which individual genomes can be projected, akin to the standardised 3-dimensional brain space used in the neuroimaging community [21]. 2. An updated genomic reference is likely to remain the best reference space for mapping and viewing transcriptomic data, as well as information about DNA (and RNA) modifications as these emerge. *Transcriptomic annotations:* 3. It would be extremely useful for future annotations to flag whether a given transcript has been derived from computational reconstruction or has been directly sequenced either using long-read nucleic acid sequencing approaches or supported by a complete peptide sequence (see points 5 and 6), producing ‘predicted’ and ‘high confidence’ sets, respectively. These complementary annotations could be considered somewhat separate (analogous to the current manual vs. automatically derived transcriptomic annotations) allowing researchers to use whichever best suits their needs and aiding harmonisation with protein annotations (see points 5 and 6). 4. Current transcriptomic annotations are assembled largely based on nucleic acid sequences. They therefore miss out on corroborating information from orthogonal sources, including proteomic sequencing. Future annotation pipelines would benefit from the inclusion of an increased diversity of input information sources. *Protein annotations:* 5. Peptide sequence data need to be readily available if they are to feed into transcriptomic annotations. However, although attempts have been made to collate peptide information [22], no centralised repository of peptide sequence data currently exists. Instead, researchers undertaking protein sequencing typically deposit raw spectral data. Thus, there is a need for a centralised repository of peptide sequence information to allow harmonisation with other data sources. This database would either need researchers to submit sequence information or would need to derive sequences from spectral data. Notably, in both cases this would require curators to set quality control thresholds and, potentially, to derive ‘high confidence’ and ‘low confidence’ peptide sets (akin to the complementary transcriptomic annotations proposed in point 3). 6. The current Uniprot annotation focuses on producing a single record of full-length protein sequences relying heavily on manual curation. This allows the inclusion of an ‘annotation score’ giving a measure of confidence that a record is accurate. However, the challenge of producing ‘full length’ sequences from often partial sequence data applies equally to peptide sequences as nucleic acid sequences. Indeed, in the case of data derived from approaches employing tryptic digestion, the peptides are mostly short and overlap between peptides only occurs in cases of incomplete trypsin cleavage making de novo reconstruction more difficult than for short-read RNA-seq data. Note that even the highest Uniprot annotation score—“Experimental evidence at protein level”—does not guarantee that the individual sequences are accurate. As for transcriptomic annotations (point 3), confidence in the accuracy of individual sequences will be maximised by harmonising across orthogonal sources of information. It will likely be appropriate to move from a single annotation to the production of multiple protein annotations (e.g. ‘high confidence’ and ‘predicted’ set) to allow researchers to select the most appropriate annotation for their needs. *Scientific literature:* 7. Harmonisation of annotations with the scientific literature (e.g. in the context of exon naming highlighted in the main text) is challenging. To facilitate the uptake of transcriptomic information in functional studies it would be beneficial for existing protein records to include additional data from transcriptomic annotations. For example, peptide sequences could be annotated with the locations of exon boundaries and the Ensembl IDs of these exons to increase the usage of standardised naming by those conducting functional studies. Furthermore, it may be useful to allow the direct submission of additional (non-sequence) information by the community (e.g. the ‘colloquial’ names for individual exons) to allow this information to ‘flow’ backwards into nucleotide and protein annotations thereby improving their consistency with the wider scientific literature. 8. Reporting guidelines should be developed and mandated by publishers to standardise nomenclature across the different fields and minimise ambiguity in publications.	
